# Bronchogenic Cyst as an Unusual Cause of a Persistent Cough and Wheeze in Children: A Case Report and Literature Review

**DOI:** 10.1155/2018/9590829

**Published:** 2018-02-21

**Authors:** Ahmed Abushahin, Abdulla Zarroug, Magda Wagdi, Ibrahim Janahi

**Affiliations:** ^1^Division of Pediatric Pulmonology, Hamad Medical Corporation, Doha, Qatar; ^2^Department of Pediatric Surgery, Sidra Medical and Research Center, Doha, Qatar; ^3^Division of General Pediatrics, Hamad Medical Corporation, Doha, Qatar

## Abstract

Wheezing and cough are common case scenarios that pediatricians encountered in their office practices. Although a bronchogenic cyst is an uncommon condition, it is essential to be considered in the differential diagnosis of a chronic cough and wheezing among young children who fail to respond to appropriate medical treatment. A 28-month-old girl was referred to our pediatric pulmonology clinic with persistent symptoms of a cough and wheeze unresponsive to standard asthma therapy. This presentation prompted us to undertake a detailed diagnostic evaluation. The evaluation exposed a cystic mass in the middle mediastinum compressing the trachea and left main bronchus. The cyst was excised and confirmed pathologically to be a benign bronchogenic cyst. Subsequently, the patient recovered well and had been free of respiratory symptoms during follow-up visits. This report highlights one of the rare causes of wheezing and cough in young children and emphasizes the importance of considering it in the differential diagnosis of a child presenting with refractory asthma-like symptoms. This is important for early diagnosis and management and to avoid unpredictable complications of this treatable condition.

## 1. Introduction

The common symptoms of asthma in children include a cough, wheeze, and breathing difficulties. If these symptoms persist over a period of asthma therapy or the symptoms are not typical for asthma, additional evaluation is strongly recommended.

Bronchogenic cysts (BCs) are a rare congenital malformation of the foregut that is typically found in the mediastinum [1]. Affected patients can present with symptoms that are related to their compressive effect and irritation of the surrounding structures [2].

We report on a child who presented with persistent wheeze and cough, not responding to medical treatment. Further investigation revealed that the cause of this child's symptoms and signs was a bronchogenic cyst.

## 2. Case Presentation

A 28-month-old girl was referred to our clinic because of a persistent cough, wheeze, and increased work of breathing for the preceding three months. Despite oral antibiotics and high doses of inhaled corticosteroids prescribed by her primary physician, cough and wheeze were persistent. Parents denied any history of witnessed foreign body aspiration, and there were no feeding-related symptoms. The patient was born at term with an uneventful postnatal course. Her history was significant for episodes of cough and intermittent wheeze that begun at one year of age; these episodes were usually treated with bronchodilators and inhaled steroids. There was no history of atopy in the family.

Initial physical examination revealed normal growth parameters, a respiratory rate of 40 breaths/minute, and no retractions. Oxygen saturation ranged from 94 to 95% on room air, and she was afebrile. Lung auscultation revealed decreased air exchange in the left lung; the rest of her physical examination was normal.

**Figure 1:**
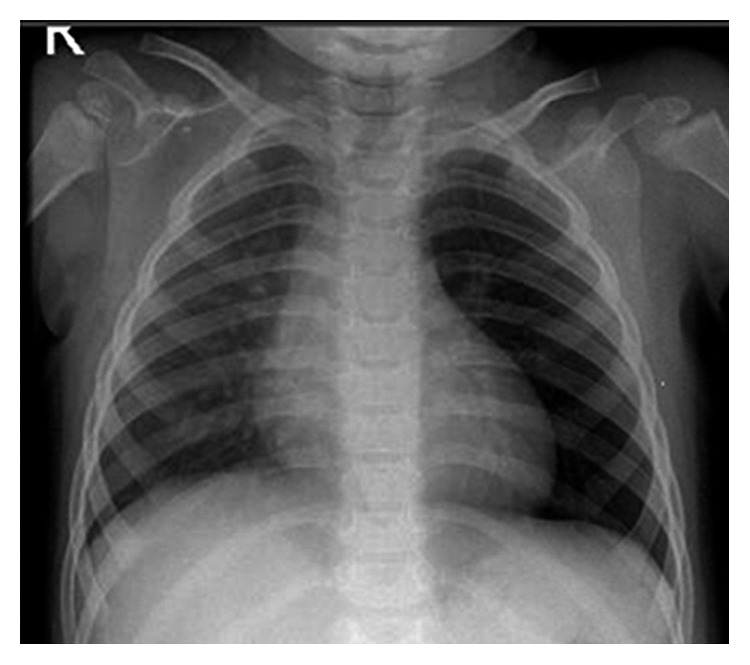
Chest X-ray showing hyperinflation on the left side.

A chest X-ray was performed for abnormal breath sounds and showed hyperlucency of the left lung (Figure 1). A barium swallow study was normal with no apparent external indentation or displacement of the esophagus and no evidence for GERD.

Blood work, including routine hematology and biochemistry tests, was within normal limits. Results of virology PCR for CMV, EBV, and adenovirus were negative. Acid-fast bacilli smear, culture, and TB PCR were negative.

**Figure 2:**
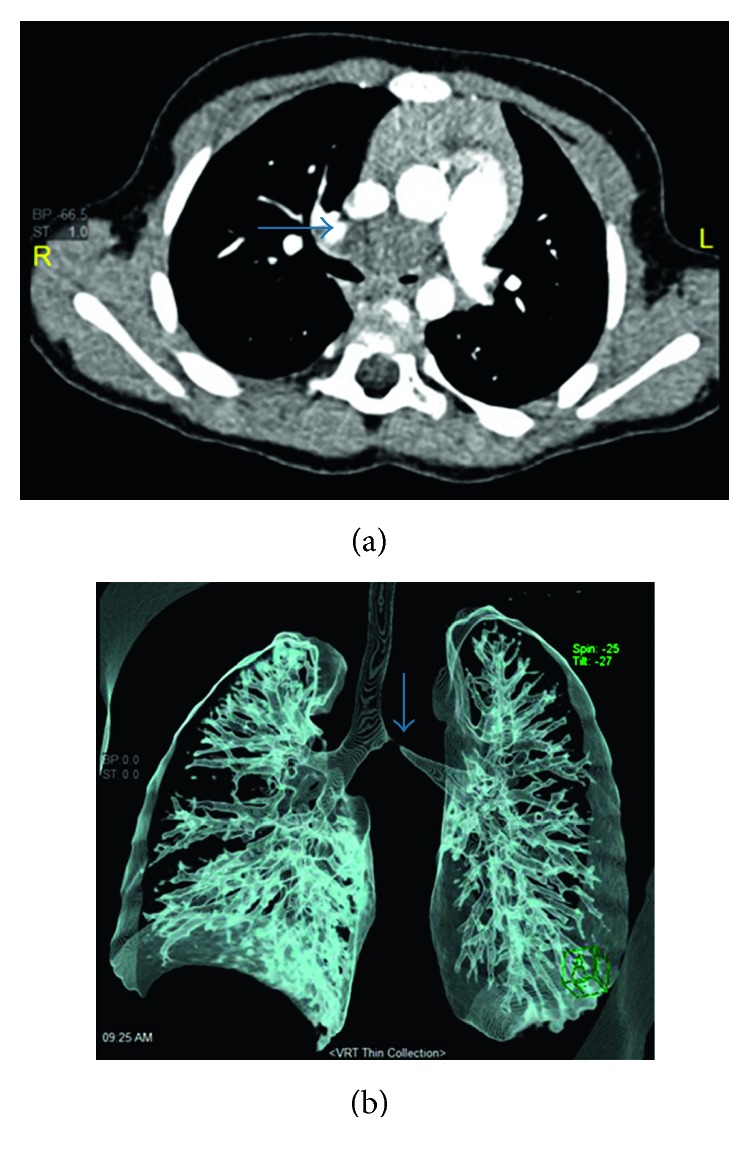
(a) CT scan of the chest showing a hypodense cystic lesion in the middle mediastinum (arrow), causing compression on the carina and the proximal aspect of the mainstem bronchi, more pronounced on the left main bronchus. (b) 3D reconstruction of the CT scan showing a significant narrowing of the left main bronchus (arrow), caused by external compression of the adjacent cystic lesion.

**Figure 3:**
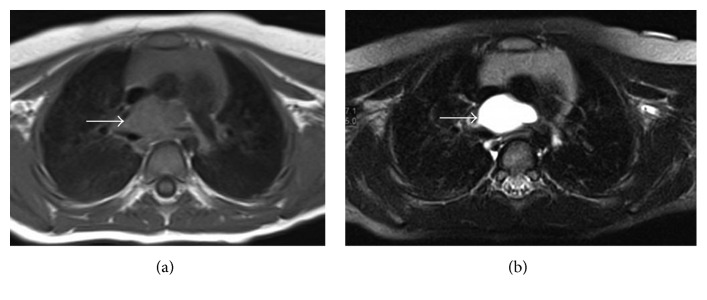
MRI of the chest showing lobulated mass lesion heterogeneously hypointense on T1-weighted images (arrow) (a), and hyperintense on T2-weighted images (arrow) (b). The mass is in the middle mediastinum, at the level of the carina and adjacent to the trachea extending to the subcarinal region, causing compression on the carina and both main bronchi, more pronounced on the left main bronchus.

On flexible bronchoscopy examination of her lower airways, no foreign body was seen, but significant external nonpulsatile compression over the left main bronchus was noticed; the rest of the airway anatomy was normal. This finding prompted us to undertake a detailed evaluation. Thoracic computed tomography (CT) scan and angiogram showed a soft tissue mass in the middle mediastinum, compressing the carina and proximal part of the right and left main bronchi, more pronounced on the left main bronchus (Figure 2). This mass was further delineated by magnetic resonance imaging of the thorax with contrast which revealed a lobulated cystic mass lesion (Figure 3).

These findings were in favor of the diagnosis of a bronchogenic cyst in the middle mediastinum. Therefore, the patient underwent thoracotomy and surgical removal of a cystic mass which was found during surgery to be adjacent to the posterior trachea and left main bronchus. Pathological examination of the mass showed a cystic lesion lined by the ciliated columnar epithelium surrounded by a fibromuscular wall containing the cartilage and nests of bronchial submucosal glands, confirming the probable diagnosis of a bronchogenic cyst. There was no evidence of malignancy.

The postoperative course was uneventful. The patient had been free of any respiratory symptoms and signs, including a cough and wheezing in the subsequent follow-up visits.

## 3. Discussion

This child presented with a persistent cough and wheeze that were not responding to standard asthma treatment. We considered the causes of wheezing and coughing in children. Therefore, she underwent flexible bronchoscopy, CT scan, and magnetic resonance imaging (MRI) of the chest that revealed a bronchogenic cyst in the middle mediastinum. Surgical removal of the mass was successfully performed, and pathologic examination confirmed the diagnosis of the bronchogenic cyst.

Bronchogenic cyst (BC) is a rare congenital pulmonary malformation with a prevalence of 1 : 42,000–1 : 68,000 [3]. BCs constitute 13–15% of the congenital cystic lung diseases and 6% of the mediastinum masses in children [4, 5]. Abnormal budding of the ventral foregut leads to the formation of BC anywhere along the respiratory system or esophagus. The location of BC formation can vary depending on the time of their origin [6, 7]. Up to 85% are in the mediastinum, and 12% are parenchymal in origin involving primarily lower lobes. However, unusual locations like the neck, pericardium, or abdomen have been reported [1, 7, 8]. In our patient, BC originated from the trachea in the middle mediastinum.

Ribet et al. reported that BCs were symptomatic in 70.8% of affected children [6]. The symptoms are usually secondary to either the compressive effect of the enlarged cyst on the adjacent structures or infection in the cyst. The former is more common in infants and young children presenting as a retraction, cough, and wheeze, while the latter is more common in adults presenting as recurrent pneumonia [4, 6, 9]. In rare conditions, mediastinal BC can present in the neonatal period with severe respiratory distress and stridor [10]. However, about one-third of BCs are asymptomatic and are found incidentally in adults and some children [2]. In our patient, obstructive symptoms were not present in the neonatal period, but ultimately, she became symptomatic.

Diagnostic studies include chest X-ray, barium swallow, bronchoscopy, and CT scan/MRI of the chest. A chest radiograph is diagnosed in only 77% of cases [11]. Usually, they reveal well-defined homogeneous density, tracheal deviation, and a compression effect-like atelectasis or emphysema. Flexible bronchoscopy may reveal airway compression, and barium swallow may show an indentation [6]. CT scan/MRI of the chest is extremely valuable for accurate diagnosis, defines the anatomy, and rules out a list of differentials that include cystic hygroma, lymphangioma, neuroenteric cyst, esophageal duplication cyst, and thymic cyst [1, 4, 9]. Ultrasonography makes the antenatal diagnosis with a reported accuracy in more than 70% of cases [12]. In our patient, the cyst was not visible on plain film; it only showed hyperinflation of the left lung. The bronchoscopy examination drove us toward the diagnosis. The CT scan and the MRI of the chest were highly suggestive of a bronchogenic cyst. In almost all cases, pathological studies are required for a definitive diagnosis. In our patient, the microscopic findings revealed a benign bronchogenic cyst. In rare cases, reports of malignant transformation, including pulmonary blastoma, rhabdomyosarcoma, and bronchoalveolar carcinoma, have been found in the resected bronchogenic cyst in adults (0.7%) and children [13]. To prevent further complications and to avoid the possible risk of malignant changes, complete surgical removal of the BC is strongly recommended even in asymptomatic individuals [4, 6].

In conclusion, although a chronic cough and wheeze are common symptoms of asthma in children when these symptoms do not respond to conventional treatment strategies, this should prompt the clinician to seek further investigations looking for other causes of persistent “asthma-like symptoms.” Bronchogenic cysts are an uncommon developmental anomaly of the tracheobronchial tree, which may cause airway compression and mimic asthma. Pediatricians should consider bronchogenic cyst as an important asthma masquerader, particularly in young children who have persistent symptoms and inadequate response to asthma therapy. Surgical resection remains the treatment of choice in all cases to confirm the diagnosis and to prevent unpredicted complications.

## Abbreviations

BC: Bronchogenic cyst
GERD: Gastroesophageal reflux
PCR: Polymerase chain reaction
CMV: Cytomegalovirus
EBV: Epstein-Barr virus
TB: Tuberculosis
Chest CT scan: Chest computed tomography scan
Chest MRI: Magnetic resonance imaging of the chest.
